# Hygrothermal Degradation of Epoxy Electrical Insulating Material—Testing and Mathematical Modeling

**DOI:** 10.3390/polym16142026

**Published:** 2024-07-16

**Authors:** Jan Leffler, Jan Kaska, Petr Kadlec, Pavel Prosr, Vaclav Smidl, Pavel Trnka

**Affiliations:** 1 Department of Materials and Technology, Faculty of Electrical Engineering, University of West Bohemia, 301 00 Pilsen, Czech Republic; 2 Department of Electrical and Computational Engineering, Faculty of Electrical Engineering, University of West Bohemia, 301 00 Pilsen, Czech Republic; kaskaj@fel.zcu.cz; 3 Research and Innovation Centre for Electrical Engineering, Faculty of Electrical Engineering, University of West Bohemia, 301 00 Pilsen, Czech Republic

**Keywords:** multi-factor stress, hygrothermal degradation, epoxy resin, material testing, degradation models, Bayesian experimental design

## Abstract

The degradation of electrical insulating materials has been a subject of interest for decades as they are commonly applied in many fields of electrical engineering. Suitably modeling such a process is important since the known and well-described degradation process reveals the effect of ambient conditions, and this allows us to possibly estimate a material’s remaining useful life. However, not many studies are dealing with the effect of the hygrothermal degradation of impregnating mono-component epoxy resins in the context of electrical engineering. Therefore, this study deals with this issue and discusses both the dielectric response (based on the measurement of relative permittivity, dissipation factor, and dielectric strength) and the mechanical response (based on measurements of tensile strength and Shore D hardness) to a hygrothermal degradation experiment. In addition, the results of thermal analyses are presented for the evaluation of the pristine specimen manufacturing process and possible post-curing processes. Furthermore, this study presents several methodologies for modeling the degradation process, including a novel methodology in this area based on Bayesian experimental design. As an outcome, mechanical parameters are proven to be specific in terms of the actual condition of the material and the Bayesian enhanced degradation model seems to be superior to the conventional evaluation methods in this particular study.

## 1. Introduction

An electrical insulation system (EIS) is an important component in all types of conventional and modern designs, for both rotating electrical machines and transformers [1,2,3]. This is documented in many publicly available studies that present the fact that the downtime of an electrical machine is commonly associated with the presence of an EIS and bearings in general failures. Of course, the majority of main failure modes and further specific failure-related mechanisms and locations depend on power and voltage ratings and on the particular type of machine, as this might include transformers [4,5,6,7], low-voltage through high-voltage motors [8,9,10], and alternators [11,12]. Thus, it is important to study the relevant effects that degrade such a machine or its specific elements or material. These might be generally categorized as thermal, electrical, mechanical, and ambient degradation factors. The degradation of a material under operational conditions considering the mentioned factors might be present at both the nominal and elevated levels. The monitoring of such deterioration provides useful information about machine service life, and this is often subsequently evaluated in order to estimate the remaining useful life (RUL) and to diagnose a device. Thus, many approaches, strategies, and Condition Monitoring and Diagnostics (CMD) systems, along with data analyses, have been discussed and developed [13,14,15]. These are beneficially utilized in asset management frameworks as machines usually become significant elements in an asset portfolio. Moreover, an aging model is important for consumed lifespan determination, and thus it allows an asset owner to schedule and execute maintenance tasks and to select an appropriate maintenance strategy according to the specific application. Considering the knowledge of degradation or lifespan models, these strategies are mainly (i) the Condition-based Maintenance (CbM) approach and (ii) the Predictive Maintenance (PdM) approach (sometimes interchanged or associated depending on the author [16,17]), which allow effective failure occurrence mitigation or even elimination. This enables an asset owner to save a significant amount of resources associated with failure-oriented or maintenance-oriented downtime and to optimize the lifespan utilization of an asset and linked logistics, and thus to contribute to a more sustainable approach in their industry [17,18]. The aforementioned matter is one regarding wear-out failures, which can be predicted via models directly in the time domain at each time instant. On the other hand, abrupt failures have to be modeled statistically based on data from a large series of assets with very similar or identical parameters, which is beyond the scope of this study.

Many approaches to these issues have been used in the past. For instance, (i) empirical (physically informed empirical relations) and physical aging models [1,19,20,21,22,23], (ii) the modeling of failure probability based on statistical data obtained through the failure data of large series of similar machines or components and using statistical distributions or tools such as Markov chains or Wiener and Gamma processes [20,21,22,23,24], and (iii) the design of experiments along with Response Surface Methodology (RSM) as a special case of regression-based model based on empirical observation [25,26,27]; also, in the past few years, (iv) machine learning has been vastly incorporated [22,23,28,29], especially using Artificial Neural Networks (ANNs) and Support Vector Machines (SVMs) [23]. The use of hybrid methods should also be pointed out as an approach that utilizes the advantages of the respective approaches [23]. A machine learning approach is often used in component and material diagnostics for pattern recognition, most often using Neural Networks [15,30]. However, since the aim of this study was to find a relatively simple material model, the Bayesian model selection method was used. Although this method is not fundamentally new and is already utilized in degradation experiments [31,32,33], it is not widely used in power electrical engineering and technical diagnostics as it has emerged only recently [18], and it is not yet used for this particular type of material and multi-factor degradation. Usually, the various electrical insulation materials and composites in real applications are aged by several factors at once, including hygrothermal degradation. Thus, such an experiment might be time-, device-, and material-demanding. Nevertheless, the published studies on the topic of hygrothermal degradation often provide the structural or another response of a novel or modified material to degradation factor exposure, or possibly an investigation of related parameters while not including a degradation model itself [34,35,36].

**Figure 1:**
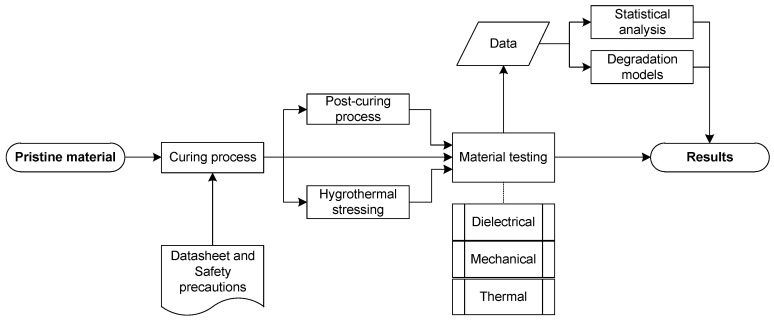
The study overview diagram.

The main objective of this study is to present the results of material testing and to develop a new methodology for a one-factor or multi-factor degradation assessment of electrical insulating materials via a mathematical model in general. This methodology should, in addition to the mean, return the variance in measurements and allow for the optimal design of experiments to find the most probable model as quickly as possible. This is achieved through the probabilistic approach to models linear in their parameters and further Bayesian model selection. This approach allows one to evaluate the variance, discriminate between models, and optimally design the experimental space, and it seems to be superior to the conventional regression methods used in this study. For these purposes, hygrothermal degradation was selected in order to collect data for the model and to compare its predictions with those obtained through the selected conventional methods. The hygrothermal stressing experimental space was divided according to the design of experiments (DoE) for a two-level two-factor experiment with a first-order Response Surface Methodology (RSM) model with an interaction [25]. Along with this aim, we introduce not only thermal analysis results revealing properties of the evaluated material but also selected dielectric and mechanical properties of the investigated material before and after the degradation process to demonstrate the material behavior. Figure 1 presents an overview of the whole study as it is quite complex.

## 2. Materials and Methods

In this study, hygrothermal degradation was selected for the material deterioration. Hence, the simultaneously acting degradation factors were temperature and relative humidity in the designed experiment. The manufacturing process of the specimens was documented using thermal analyses and the response to the stress was subsequently evaluated.

### 2.1. Material

An unmodified impregnating mono-component epoxy resin for insulation systems of low-voltage rotating machines, transformers, and coils was selected for this experiment. This epoxy resin is based on bisphenol A and epichlorohydrin. Its viscosity according to the datasheet is 400 ± 100 mPa·s at 23 °C. The curing of this one-component epoxy resin (consisting, according to the manufacturer’s information, of substances with CAS 25068-38-6, CAS 2425-79-8, and CAS 68609-97-2) involves the opening of the oxirane rings at a high temperature (140 °C to 160 °C) by a nucleophilic reaction of the hydroxyl groups with the epoxy groups to form secondary alcohol, which can further react with the epoxy groups. The diepoxide monomer causes crosslinking and the monoepoxide monomer ensures the formation of linear chains. This reaction takes place at the aforementioned high temperature.

### 2.2. Preparation of Experimental Specimens

First of all, it was necessary to establish a stable manufacturing procedure consisting of 15 min of vacuuming the uncured liquid epoxy within a filtering flask in order to significantly reduce the air content that leads to cavity creation in the cured samples. Then, molds for gravitation casting were used, followed by another 5 min in the vacuum chamber (0.1 bar). The molds were subjected to drying at 60 °C for at least 24 h. The epoxy specimen curing was carried out following one of the manufacturer’s recommended procedures under temperature conditions of 140 °C for 6 h. Eventually, no post-curing process was applied in the experiment.

### 2.3. Degradation Experiment Setup

Firstly, several batches were manufactured in order to (i) set the baseline for selected parameters of the observed material and (ii) remove the flaws in the production process of the samples. The number of air cavities was minimalized.

Next, the points in the experimental space were designated under climate chamber limitations. The high and low levels of the selected degradation factors were set according to Table 1, along with an exposure time of 336 h. The settings were subsequently compared to similarly oriented published studies [35,36,37].

The glass transition temperatures were determined via thermal analyses and similar epoxy resins were also taken into account. Another limiting factor was the operational range of the climate chamber VC 7018 (Vötsch, Balingen, Germany) which is defined as 95 °C with 95% relative humidity. Eventually, the limits were selected as 80 and 95 °C for the temperature and 55 to 95% for relative humidity. The exposure period was selected as 336 h for this setup.

### 2.4. Measurements and Analyses

The epoxy resin used for this hygrothermal degradation experiment was firstly characterized in a pristine state through selected thermal analyses, dielectric measurements, and mechanical tests. Thermal analyses were also used to evaluate the degree of curing achieved via the manufacturer’s curing procedure (140 °C for 6 h). Subsequently, the consequence of hygrothermal degradation was evaluated using dielectric measurements and the results of mechanical tests. The selected thermal analyses included differential scanning calorimetry (DSC), recording the heat flow through the specimen, and simultaneous thermal analysis (STA), combining the recording of the heat flow through the specimen with the recording of the mass change (thermogravimetry) during a controlled temperature program. Another thermal analysis used was the dynamic mechanical analysis (DMA), chosen to evaluate the response of the epoxy resin to mechanical harmonic loading over a wide temperature range based on the mechanical loss factor. The realized dielectric measurements are primarily measurements of relative permittivity, dielectric loss factor, and dielectric strength. Mechanical tests included a tensile test and a hardness test based on the Shore method.

DSC was performed using a DSC Q2000 apparatus (TA Instruments, New Castle, DE, USA). The measurements were carried out in nitrogen with a volume flow rate of 50 mL/min. Specimens of 9 mg were prepared for testing and analyses, which were carried out at temperatures ranging from 0 to 280 °C with a heating and cooling rate of 10 °C/min (further specified in the Section 3). STA was carried out on an SDT Q600 apparatus (TA Instruments) in the air at a volume flow rate of 100 mL/min. The STA was performed at temperatures ranging from ambient to 700 °C with a heating rate of 10 °C/min for each specimen of 9 mg. DMA was performed using a DMA Q800 (TA Instruments) in the air atmosphere. Rectangular specimens with dimensions of approximately 35 × 9 mm were tested using DMA with dual cantilever clamps. The oscillation amplitude was set to 50 μm and the frequency was set to 1 Hz, and the temperature ranged from laboratory ambient temperature to 150 °C, with a heating rate of 5 °C/min.

The selected dielectric properties were measured for 100×100 mm flat square solid epoxy specimens via the vector bridge Tettex 2830/2831 (Haefely, Basel, Switzerland). The applied voltage was set to 500 Vrms at a frequency of 50 Hz and the applied compressive force of the electrode system caused pressure equal to 6 N/m^2^. Measurements of dielectric parameters using the Tettex vector bridge were performed at a laboratory temperature of about 23 °C, a relative humidity of about 40%, and an atmospheric pressure of around 980 hPa. Comprehensive dielectric analysis was performed with broadband dielectric spectroscopy (BDS) using the apparatus based on Alpha-A mainframe (Novocontrol Technologies, Montabaur, Germany) with settings of 1 Vrms and electrodes of diameter 30 mm, within a variable temperature and frequency range of 0 to 120 °C and 5 · 10^−1^ to 1 · 10^6^ Hz. The dielectric strength measurements were performed via a modular system for high-voltage AC (50 Hz) ramp testing (HighVolt, Dresden, Germany) with the following settings: a voltage level of 100 kV with a test time limit of 60 s, a voltage set-point tolerance of 3%, and a voltage rise of 1500 V/s, while the primary current limit was set to 30 A. The voltage rise was kept at the same value throughout the measurements and the breakdown occurred approximately between the 15th second and the 30th second. The specimens were placed horizontally between two aligned identical cylindrical electrodes with an edge-rounding radius of 3 mm and a diameter of 25 mm according to the standard IEC 60243-1 [38].

In the field of conventional mechanical testing, tensile tests according to the standard ISO 527 [39] were performed using the LabTest 3.030 testing machine (LaborTech, Opava, Czech Republic). Dog-bone-shaped specimens of type 5A according to ISO 527-2 [40] were prepared for testing. The test speed was set at 0.1 mm/s and the initial distance between the jaws was 50 mm. This low test speed was set based on optimization measurements considering the fact that materials with high hardness and brittleness were being tested. The Shore D measurement of hardness for rigid specimens was conducted afterward.

### 2.5. Model Assembling Methods

There are various procedures for acquiring an aging or degradation model. As the authors of this work, we selected (i) a physically informed empirical relation experimental design, (ii) a Response Surface Methodology experimental design using a two-factor first-order model with an interaction, and (iii) a Bayesian experimental design.

#### 2.5.1. Physically Informed Empirical Relation

These models are based on the physical principle of a material degradation process. In some cases, direct implementation of certain physical laws is possible (e.g., Crines’ model) [19]. However, this is not usually applicable to many degradation-related issues. Thus, empirical-based physical models were employed. These models are also based on the physical principle of degradation, but the model parameters are found by regression using measured data gathered via accelerated-aging experiments [20,21].

Unfortunately, a hygrothermal degradation process description within a certain material and with a characteristic response such as a dielectric strength is quite specific demand. However, a general lifespan (τ) model of components under hygrothermal stress is proposed in [21] and can be described via the following equation:

(1) $$\tau =\theta_{1}\;\exp\left(\frac{\theta_{2}}{\vartheta }+\frac{\theta_{3}}{\mathrm{RH}}\right),$$

where $\theta_{1}$, $\theta_{2}$, and $\theta_{3}$ are parameters obtained via regression, ϑ represents the temperature, and RH stands for relative humidity.

The benefit of this approach is the fact that the procedure is quite straightforward as the selected equation for regression is applied directly and the coefficients are determined via the ordinary least-squares method. On the other hand, the more generic degradation-related equation could be considered a significant disadvantage as the estimation of the system response might be imprecise.

#### 2.5.2. Response Surface Methodology

A general approach used to assess the response of a system to a certain factor or multiple factors is the DoE with RSM [25]. In this case, this study used a two-level two-factor experimental design and a first-order RSM model. This is a well-established method for many investigation issues, e.g., for investigating the impacts of temperature and alternate voltage [26,27]. The benefits include that one does not need to know the exact system behavior and the full factorial experimental design returns the information about factors’ interactions. The essential disadvantage is the poor scalability of this procedure for a high number of factors. Hence, for extensive experiments, it is advised to perform a pre-experiment to determine which factors and interactions are important and which are not. To make an experiment less demanding, many reduction methods might be employed, e.g., the Taguchi or Shainin method [41] or the fractional factorial design (excluding selected higher-order interactions) [25].

Once all values are collected, the following general two-factor first-order model equation can be used:

(2) $$Y_{ijp}=\mu +a_{i}+b_{j}+t_{ij}+e_{ijp}.$$

In Equations (2) through (5), *Y* is the system’s response to the factors, μ is the mean value, *a* and *b* are elements containing the factor level (*F*s and *I*) and the effect strengths (*E*s) according to Equation (3), and, similarly, *t* represents the same, but for interactions between the factors. The element *e* represents the residual least-squares error, which is constant and might be caused by other sources of variability, for instance, measurement variability, background noise, and other unknown influences. In general, the indexes *i* and *j* stand for the possible applied multiple levels of acting factors, and the index *p* respects the number of possible replications on the same factor level.

After completion of all of the respective experiment runs, it is possible to obtain each effect strength as follows:

(3) $$\begin{array}{cc} E_{A} & =\frac{-y_{1}+y_{2}-y_{3}+y_{4}}{4}=\frac{a_{i}}{F_{A}},\\ E_{B} & =\frac{-y_{1}-y_{2}+y_{3}+y_{4}}{4}=\frac{b_{j}}{F_{B}},\\ E_{\mathrm{AB}} & =\frac{y_{1}-y_{2}-y_{3}+y_{4}}{4}=\frac{t_{ij}}{I_{\mathrm{AB}}}. \end{array}$$

These equations can be more conveniently rewritten in a matrix notation, wherein the matrix that respects the experimental setup is denoted as D:

(4) $$E=D^{-1}Y,$$

which can be rewritten as

(5) μEAEBEAB=14·−1−1−1−1−1+1−1+1−1−1+1+1+1−1−1+1·y1y2y3y4.

**Figure 2:**
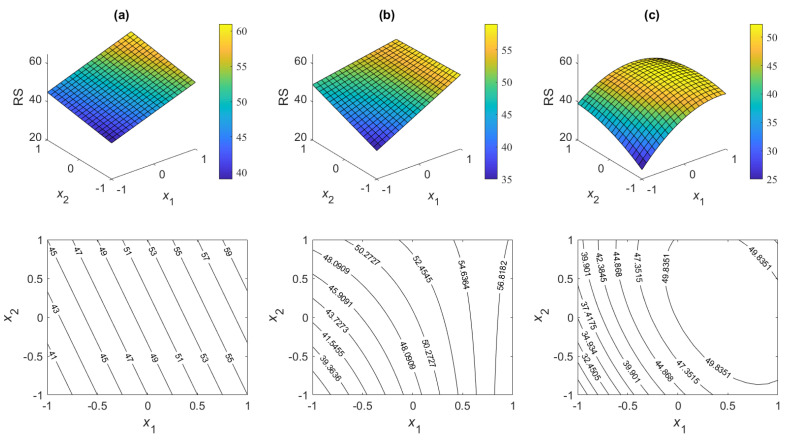
RSM contour plot result examples: (**a**) two-factor first-order model; (**b**) two-factor first-order model with an interaction; (**c**) two-factor second-order model with interactions.

An example of the response of such a first-order model with interaction can be seen in Figure 2b, among others [25]. The *z*-axis together with the label “RS” represents the respective response surfaces.

#### 2.5.3. Bayesian Experimental Design

The empirical or physical approach introduces the model to describe degradation when the process is roughly known. On the other hand, the RSM approach suggests a very general procedure in order to describe any experiment results. If the empirical or physical model is linear in its parameters, then the regression coefficients can be easily found using the ordinary least-squares (OLS) method. However, it is not always clear from the beginning which model and which approach will be suitable. Comparing models based on the mean squared error or coefficient of determination $R^{2}$ can then be burdened with the problem of overfitting. The Bayesian approach allows the model to be considered in probabilistic terms, and so the probabilities of the different models can be compared. Moreover, it is possible to design the experiment so that the most likely model is found with the fewest number of measurements. We selected four models, wherein the predicted value is the logarithm of the dielectric strength $y=\ln(E_{p})$ and the basis functions are as follows:

(6) $$\begin{array}{cc} M_{1}: & \phi_{1}\left(x\right)=\left[1,\;\vartheta ,\;\mathrm{RH}\right],\\ M_{2}: & \phi_{2}\left(x\right)=\left[1,\;\vartheta ,\;\mathrm{RH},\;\vartheta \;\mathrm{RH}\right],\\ M_{3}: & \phi_{3}\left(x\right)=\left[1,\;\vartheta ,\;\mathrm{RH},\;\vartheta^{2},\;{\mathrm{RH}}^{2}\right],\\ M_{4}: & \phi_{4}\left(x\right)=\left[1,\;\vartheta ,\;\mathrm{RH},\;\;\vartheta^{2},\;{\mathrm{RH}}^{2},\;\vartheta^{2}\;{\mathrm{RH}}^{2}\right]. \end{array}$$

Bayesian linear regression [42] further generalizes the least-squares method by not searching a single value of parameter estimate $\hat{\theta }$, but instead providing a full probability distribution of all possible values given in the data, p(θ|D). This approach is closely related to the OLS formulation when both the likelihood function and the prior have Gaussian form:

(7) $$\begin{array}{cc} p(y|\theta ,x) & =N(\theta^{⊤}\Phi \left(x\right),\sigma_{D}^{2}I), \end{array}$$

(8) $$\begin{array}{cc} p(\theta ) & =N(\theta_{0},\sigma_{0}^{2}I), \end{array}$$

where N denotes the multivariate Gaussian distribution with the mean value and covariance matrix as arguments, and *I* is the identity matrix of appropriate dimensions. The prior parameter $\theta_{0}$ is chosen to be the vector of zeros, and $\sigma_{0}$ will be estimated from the data. The mean value is then the model $\theta^{⊤}\Phi \left(x\right)$, where θ represents the unknown parameters and Φ(x) is a vector of the basis functions. The posterior distribution is then computed via the Bayesian theorem

(9) p(θ|D)∝(y|θ,x)p(θ),

where $D={\left\{x_{i},y_{i}\right\}}_{i=1}^{n}$ represents the measured data. The posterior distribution again has a Gaussian form, $p\left(\theta |D\right)=N\left(\bar{\theta },S\right)$:

(10) $$\begin{array}{cc} \bar{\theta } & =\frac{1}{\sigma_{D}^{2}}S\Phi {\left(x\right)}^{⊤}y,\\ S^{-1} & =\frac{1}{\sigma_{0}^{2}}I+\frac{1}{\sigma_{D}^{2}}\Phi {\left(x\right)}^{⊤}\Phi \left(x\right). \end{array}$$

Note that for $\sigma_{0}\to \infty$, the mean value $\bar{\theta }$ corresponds to the OLS solution. The key distinction between this method and the OLS method is in the predictive variance, i.e., the uncertainty of the predicted output for a new unseen value of the factors, specifically

(11) $$\begin{array}{cc} p(y|x,D) & =N\left({\bar{\theta }}^{⊤}\Phi \left(x\right),\Sigma \left(x\right)\right), \end{array}$$

(12) $$\begin{array}{cc} \Sigma (x) & =\sigma_{D}^{2}+\Phi {\left(x\right)}^{⊤}S\Phi \left(x\right). \end{array}$$

To distinguish between multiple models $M_{i}$ (in our case, four), we need to compute the probability that those data D were generated from this model:

(13) $$\begin{array}{cc} p(M_{m}|D) & \propto p\left(y|M_{m},\Phi_{m}\left(x\right)\right)p\left(M_{m}\right)\;\forall m=1\ldots 4, \end{array}$$

where $p(M_{m})=1/4$ is the prior probability of each of the four candidate models (6), and $p(y|M_{m},\Phi_{m}\left(x\right))$ is given by Equation (11), often evaluated in logarithm:

(14) $$\begin{array}{cc} \ln p(y|M_{m},\Phi \left(x\right))=\; & -d_{m}\ln\sigma_{0,m}+n\ln\sigma_{D,m}\\ \; & -E\left({\bar{\theta }}_{m}\right)-\frac{1}{2}\ln\left(S_{m}^{-1}\right)-\frac{n}{2}\ln\left(2\pi \right),\\ E({\bar{\theta }}_{m})=\; & \frac{1}{2\sigma_{D,m}^{2}}{∥{\hat{y}}_{m}-y∥}^{2}+\frac{1}{2\sigma_{0,m}^{2}}{\bar{\theta }}_{m}^{⊤}{\bar{\theta }}_{m}, \end{array}$$

(15) $$\begin{array}{cc} {\hat{y}}_{m}=\; & {\bar{\theta }}_{n}^{⊤}\Phi_{m}\left(x\right). \end{array}$$

Equation (13) provides the probability of the *m*th model. Since the model is uncertain, the prediction has to take this uncertainty into account and average over it:

(16) $$\begin{array}{cc} p(\hat{y}|x,D) & =\sum_{m=1}^{4}w_{m}p\left(\hat{y}|x,D,M_{m}\right), \end{array}$$

(17) $$\begin{array}{cc} w_{m} & =\frac{p(y|M_{m},\Phi \left(x\right))}{\sum_{m=1}^{4}p(y|M_{m},\Phi \left(x\right))}, \end{array}$$

where $w_{m}$ is the probability (weight) of each model. This is a mixture of Gaussian predictors (11), and is denoted as the Bayesian Model Averaging (BMA) predictor. In effect, the uncertainty about the model prediction is thus increased. The variance of the prediction (16) is as follows:

(18) $$\begin{array}{cc} \; & \mathrm{var}\left(y^{\prime }|x^{\prime },D\right)=E\left(y^{\prime }y^{\prime }\right)-E{\left(y^{\prime }\right)}^{2} \end{array}$$

(19) $$\begin{array}{cc} \; & =\sum_{m=1}^{M}w_{m}\left({\hat{y}}_{m}{\hat{y}}_{m}+\Sigma_{m}\right)-{\left(\sum_{m=1}^{M}w_{m}{\hat{y}}_{m}\right)}^{2}. \end{array}$$

Since this formula is sensitive to the choice of $\sigma_{0}$, we estimated it together with $\sigma_{D,m}$ from the data for each model using the empirical Bayes procedure [42].

As the experiments in this application domain might be very expensive, we explored the ability of Bayesian optimization to obtain as much information with as few experiments as possible. The theory of optimal design is a classical method [43] with many recent applications [31]. Formally, the next experimental point is found as a solution optimization problem:

(20) $$x^{\prime }=\arg\max_{x\in X}U\left(x|D\right),$$

where U(x|D) is a chosen utility function. A common choice of utility is the information gain; however, this is quite expensive to evaluate and various approximations can be used [31], such as predictive variance (18). The summary of the approach is described in Algorithm 1.
**Algorithm 1** Summary of the Bayesian experimental design for the selection of the optimal linear model.**Init:** Choose initial specimens $x_{\left(0\right)}=x$ and measure output variable $y^{\left(0\right)}$ using e.g., the RSM recommendation. Set iteration counter l=0.**Iterate until convergence:** 1.For each model $M_{m},m=1\ldots 4$ calculate coefficients ${\bar{\theta }}_{m}$ (10) and probability $w_{m}$ using (17) for data $D^{\left(l\right)}$ 2.On the grid of candidate points $x^{\prime }$ compute predictive variance (18) 3.Select point $x^{\prime }$ with highest variance for the next measurement, 4.Perform measurement to obtain $y^{\prime }$ and extend the data sets: $D^{\left(l+1\right)}=\left[D^{\left(l\right)},x^{\prime },y^{\prime }\right]$**Return:** most likely model and its parameters $\theta_{m}$

The main benefits of this method are as follows: The method works with not one but several candidate models that can be compared based on their probability concerning the measured data. This probabilistic interpretation then leads to the use of a Bayesian optimal design, which selects experimental setups in such a way that the different models are discriminated and the most suitable one is found as quickly as possible.

## 3. Results and Discussion

### 3.1. Measurements and Analyses

In order to become acquainted with the properties and to better understand the behavior of the tested material, several thermal analyses were carried out followed by mechanical and dielectric measurements.

#### 3.1.1. Simultaneous Thermal Analysis

**Figure 3:**
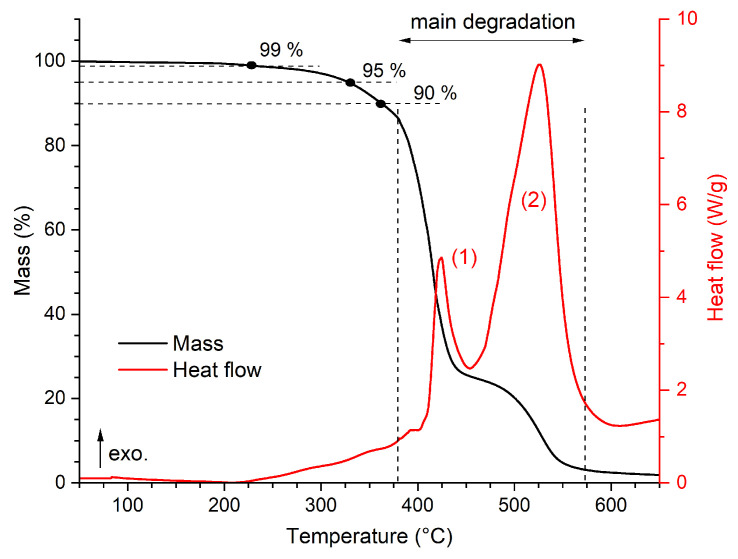
Results of the STA for the pristine state of the epoxy resin with the marked exothermic peaks.

The mass change trend obtained from the STA shows that a 1% mass loss is achieved at 227 °C, a 5% mass loss is achieved at 329 °C, and a 10% mass loss is found for a temperature of 363 °C. Up to approximately this temperature, no significant changes in heat flow are detected. From about 220 °C, only a slight thermal degradation takes place, and from about 380 °C, degradation associated with a clear exothermic process (thermo-oxidation) can be observed, which shows as the dominant peak in the heat flow trend, marked no. (1) in Figure 3, and the steepest mass loss during the analysis (steepness of about −1.2 %/°C between 380 °C and 435 °C). This is followed by the burning of decomposed epoxy resin, represented by the exothermic peak marked no. (2) in Figure 3, and a less significant mass change. The mass residue at 650 °C is less than 2%.

#### 3.1.2. Differential Scanning Calorimetry

**Figure 4:**
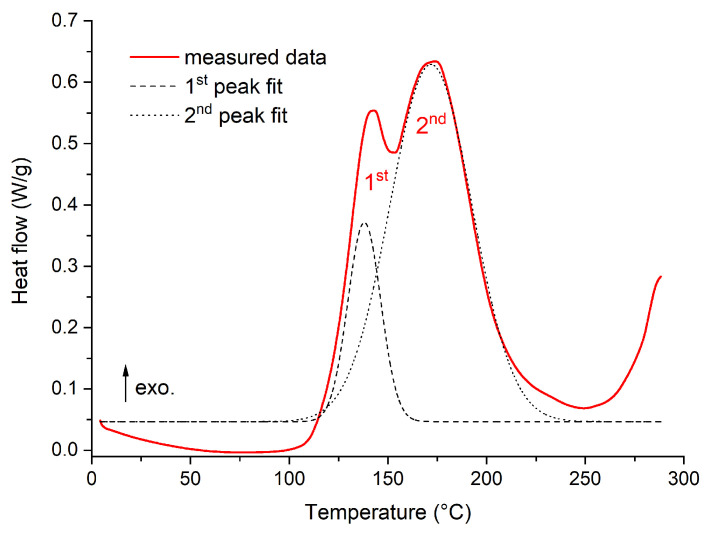
Results of DSC analyses for several thermal loading procedures.

The STA was followed by DSC analyses, which were performed over a limited temperature interval sufficient enough to assess the level of curing and to detect any additional processes in the temperature region before the occurrence of the visible heat flow change associated with degradation according to the STA results. Based on the initial analyses, it was found that the epoxy resin was not fully cured after curing at 140 °C, even after the manufacturer’s recommended time of 6 h. The identified incomplete curing was not a significant problem for further testing, but when it was detected, a more detailed analysis was carried out. Primarily, a DSC analysis was performed for the liquid uncured resin and thus the heat flow trend during curing was obtained, which is shown in Figure 4. It can be seen from the observed trend that the curing of the epoxy system occurs in two steps. The first step is characterized by an exothermic peak with a maximum at 143 °C (first peak in Figure 4). The second curing step peaks at 174 °C. The curing process can be slightly shifted based on the heating rate (here 10 °C/min), but it is clear that even with longer curing times, the material cannot be fully cured when reaching only 140 °C.

**Figure 5:**
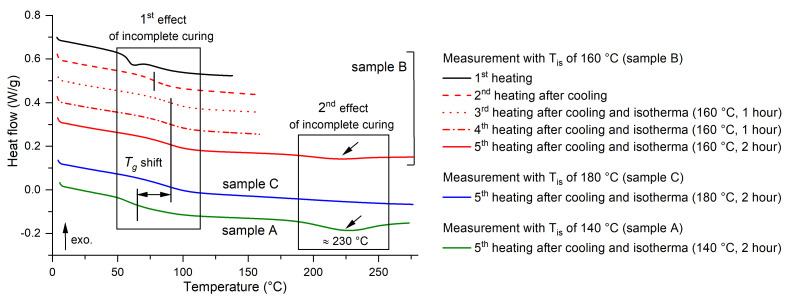
Results of DSC analyses for several post-curing procedures with the epoxy resin.

The previous assumption was further confirmed by testing a solid specimen prepared using the above procedure, which was repeatedly heated, isothermally loaded at a defined temperature, and cooled. The results from this testing are summarized in Figure 5. Overall, three sub-experiments were performed, where each specimen was always loaded in five steps. First, the specimen was heated to the curing temperature (140 °C) and then cooled (first step). Then, a second heating was implemented, and the specimen was kept at constant temperature $T_{\mathrm{is}}$ (isothermal process) for 1 h, followed by cooling (second step). This cycle was repeated two more times (third and fourth step), except that the isothermal loading in the fourth step lasted 2 h. Finally, heating to a temperature of 280 °C was carried out (fifth step). For specimen A, the $T_{\mathrm{is}}$ was 140 °C; for specimen B, this temperature was 160 °C; and specimen C was loaded to 180 °C. Figure 5 shows the complete results for specimen B and then compares the final heating to 280 °C for all three samples. The effect of curing temperature is evident from the shift in the glass transition temperature $T_{g}$, but also from the less significant endothermic peak with a maximum of approximately 230 °C. The glass transition temperature is approximately 59 °C for the first heating as well as for subsequent heatings up to 140 °C, although for the first heating, the $T_{g}$ is more difficult to determine due to the effect of enthalpy relaxation [44]. For specimen B, there is an increase in $T_{g}$ even with the second heating, which may be due to the heating of the small specimen and the stabilization associated with the removal of moisture and thermal history, which may be different for each specimen. This resulted in an increase in the glass transition temperature up to about 76 °C. Subsequent heating to 160 °C or up to 180 °C resulted in the $T_{g}$ increasing to about 87 °C. The measurement of sample C also showed a complete elimination of the evaluated endothermic peak.

#### 3.1.3. Dynamic Mechanical Analysis

**Figure 6:**
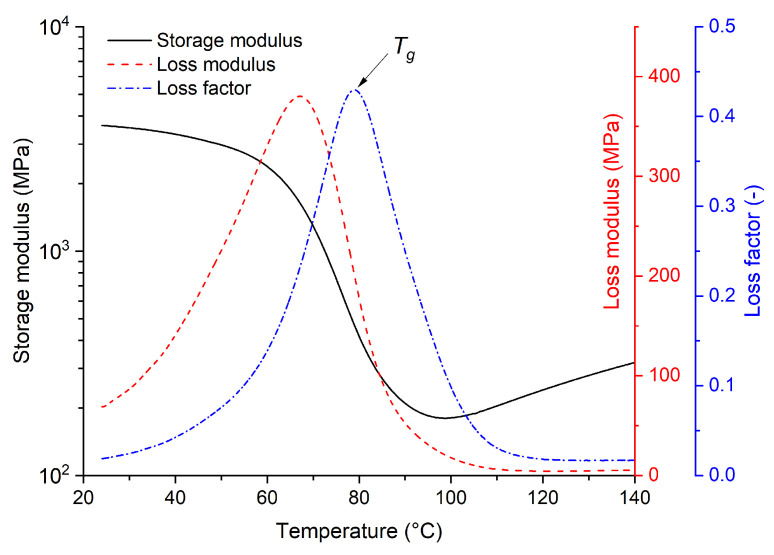
Results of DMA for the tested epoxy resin (cured at 140 °C for 6 h).

The results of the DMA of the tested epoxy resin shown in Figure 6 allow us to evaluate the behavior of the material over a wide temperature range in terms of its mechanical response. The DMA results are interpreted using three parameters: (i) the storage modulus, (ii) the loss modulus, and (iii) the loss factor. The storage modulus is a real component of the complex mechanical modulus and represents a measure of the ideal elastic resistance of the material to elastic dynamic stress at a specific frequency and temperature (characterization of the solid-to-rubber transition). The presented results show a decrease in the storage modulus of approximately one order of magnitude, indicating only a slight change in the stiffness of the material over a given temperature interval. The loss modulus is an imaginary component of the complex mechanical modulus and can be interpreted as the amount of energy that is absorbed by the specimen and converted to heat during one period of deformation, related to the mobility of the polymer chains. The maximum mobility is reached at the maximum point of the process at the glass transition temperature. The third studied parameter is the loss factor, defined as the ratio of the loss modulus to the storage modulus. The loss factor is selected to determine the glass transition temperature, and its trend approximately follows the loss modulus trend, but its maximum is shifted to higher temperatures.

From the loss factor trend, the glass transition temperature $T_{g}$ is determined as approximately 79 °C. Although this is a fundamentally different technique for determining the glass transition temperature than that used in DSC, the measured value is still very close to the value obtained for the standard cured material (140 °C, 6 h) based on DSC from the heat flow curve.

Hence, there is no point in thermal stressing below these temperatures, as the glass transition temperature $T_{g}$ is a characteristic thermoset parameter and is usually considered as the maximum service temperature of thermosets, and thus no accelerated degradation should be present [45].

#### 3.1.4. Dielectric Properties

To prove the hygrothermal degradation effect on the flat square epoxy specimens, the dielectric properties were measured before and after the applied stresses. The selected basic parameters of (i) relative permittivity, (ii) dissipation factor, and (iii) capacity were measured and analyzed at a voltage level of 500 Vrms and a frequency of 50 Hz. The mentioned parameters were evaluated for the model validation specimen before and after the hygrothermal stressing with the settings of 89.5 °C and 75.6% relative humidity for the 336 h of exposure time. The results are summarized in Table 2.

Subsequently, a BDS analysis was carried out in order to complete the characterization of the trends in the (i) relative permittivity (dielectric constant), (ii) loss factor, and (iii) dissipation factor of the tested epoxy resin over a wide frequency and temperature range.

**Figure 7:**
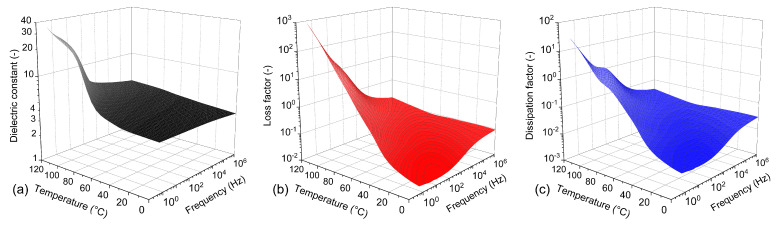
Frequency–temperature dependencies of BDS results: (**a**) dielectric constant, (**b**) loss factor, and (**c**) dissipation factor of the evaluated epoxy resin.

A comprehensive representation of the BDS results is presented in Figure 7, which shows the frequency–temperature dependence of the dielectric constant, loss factor, and dissipation factor. It is generally evident from the plotted dielectric constant dependencies that the values increase with a decreasing frequency and an increasing temperature. In the case of the dissipation factor, the trend is not so clear, with values decreasing in the mid-frequency region. However, in the frequency and temperature range used in this study, the effects of polarization processes are not clearly visible and only suggestions of less pronounced polarizations superimposed on the base conductivity component can be assumed.

**Figure 8:**
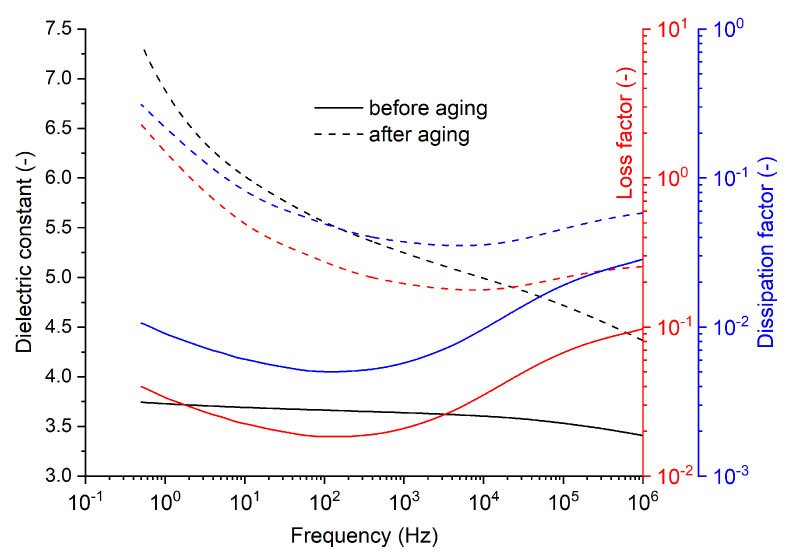
Frequency dependencies of the (i) dielectric constant, (ii) loss factor, and (iii) dissipation factor in the frequency domain at a fixed temperature of 25 °C.

Subsequently, a new batch of specimens was created according to the same manufacturing procedure. The non-aged specimen from the previous batch and the aged specimen were inspected, respectively, at a fixed temperature of 25 °C and a variable frequency through BDS. This provided information on how the mentioned parameters behave under variable-frequency conditions before and after applied hygrothermal stress. Results can be seen in Figure 8. The value of the dissipation factor obtained via BDS at 25 °C and 50 Hz is quite similar to the vector bridge measurement.

**Figure 9:**
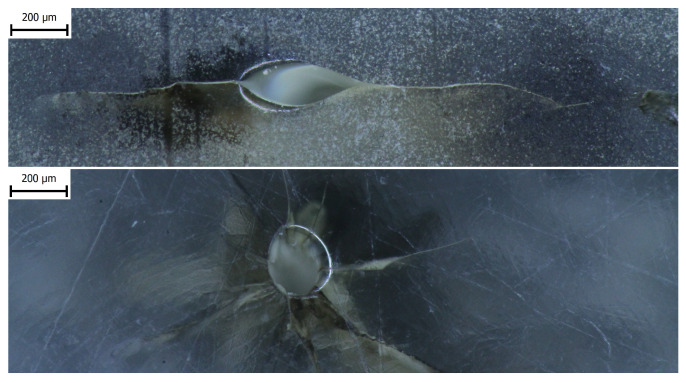
Illustrative micrographs of a discharge channel obtained via stereo microscope Olympus SZX10 after the experiment.

The dielectric strength testing was performed in order to acquire data for the degradation model’s assembly. The specimens were stored for approximately 24 h in a sealed container after the end of each experimental setup until the testing. Breakdowns were evident due to the presence of carbon in discharge channels in the solid specimens, and it was possible to localize such breakdowns afterwards. Subsequently, a microscopic inspection of the breakdown channels was carried out via a stereo microscope Olympus SZX10 (Evident Corporation, Nagano, Japan). These micrographs are presented in Figure 9.

Since dielectric strength usually has a significant variance, six specimens were stressed in each designed experimental condition. Each of the four experimental setups of these six specimens created a point in the experimental space, as can be seen in Section 3.2. Approximately five dielectric strengths were measured in each specimen in each setup, resulting in up to 30 values of dielectric breakdown. However, it should be noted that some specimens were damaged during the manufacturing process or failed to provide exactly five values of $E_{p}$, so this quantity is rather approximate.

**Figure 10:**
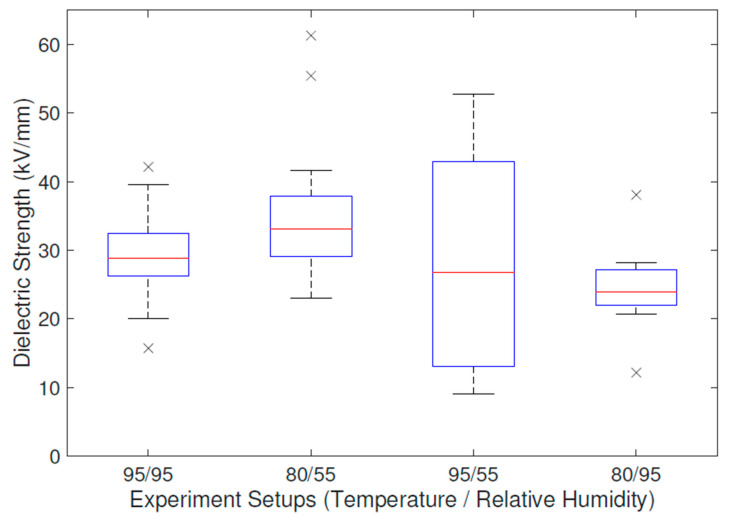
Standard boxplot of dielectric strength response to hygrothermal stress for the individual experimental runs (black crosses represent outliers).

The obtained results, presented in Figure 10, reported a high variance, so they were subjected to a one-way ANOVA at first. The null hypothesis was determined if at least one mean value differed from the others. The *p*-value was determined as $1.473\cdot 10^{-3}$, meaning that the null hypothesis could be rejected. However, not every dataset distribution was identified as the normal one. Moreover, Bartlett’s test for variance equality was subsequently carried out and the variances were evaluated as non-equal as the *p*-value was $2.495\cdot 10^{-7}$. These additional test outcomes restricted us from using the ANOVA. Thus, the Kruskal–Wallis test was applied in order to test median equality with the *p*-value $1.707\cdot 10^{-4}$, rejecting the null hypothesis. Nevertheless, the alternative hypothesis should be defined rather carefully, in the sense that at least one dataset needed to come from a different population than the others. All tests were carried out at the level of statistical significance α=0.05.

Thus, the effect of the hygrothermal degradation process on the specimens stated in publicly available studies [35,36,37] was confirmed, and the study could proceed further via different approaches to the degradation model assembly.

#### 3.1.5. Mechanical Properties

The mechanical tensile test and hardness (Shore D) measurements were performed on the prepared specimens (i) before and (ii) right after the hygrothermal stress, followed by measurements carried out (iii) after 1 day and (iv) after 4 days of relaxation in laboratory conditions.

**Figure 11:**
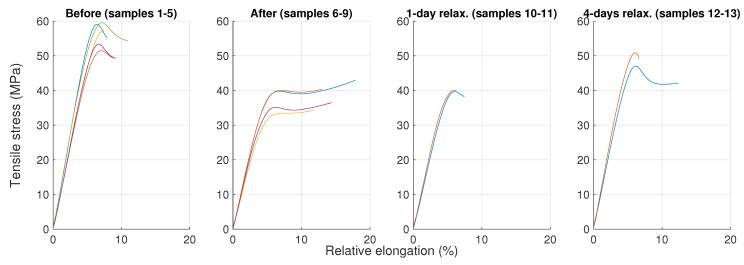
Tensile test diagrams for all tested specimens. Each line represents a measured diagram of a specimen.

**Figure 12:**
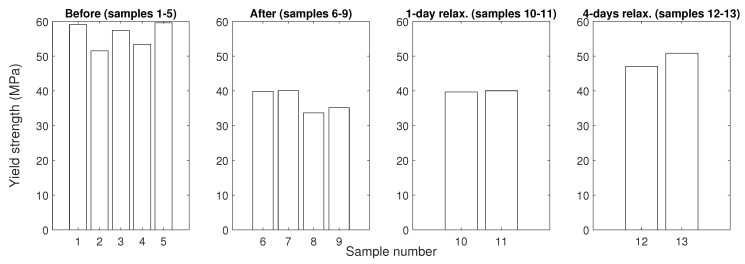
Bar charts showing the yield strengths of all tested specimens.

In Figure 11 and Figure 12, it can be observed that the epoxy resin material tended to recover its original mechanical properties in this particular case. This is probably associated with the process of desorption [46,47,48]. However, the material did not fully recover even after a 4-day relaxation period under usual ambient laboratory conditions, as the free water could be removed relatively easily [48]; however, the completion of desorption might require elevated temperatures or/and a desiccator with silica gel, as suggested, for instance, in [46,48]. Moreover, the wet specimens measured right after the exposition reported a flattened shape of the curve. Both a drop in tensile strength and a flattened shape are also reported, for instance, in [49].

**Figure 13:**
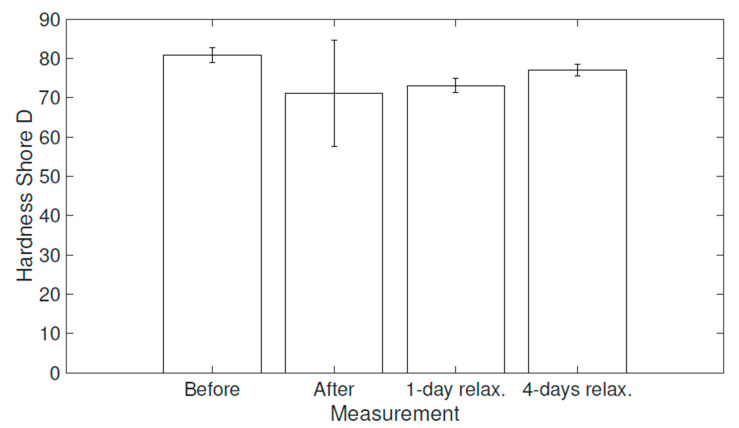
Bar chart showing the mean value of Shore D hardness and standard deviations.

This kind of behavior was also reported during the material hardness measurements using a Shore Durometer (Shore D: hard materials). The hardness tests were performed within the same time periods of relaxation used for the tensile strength measurements, and each measurement consisted of ten values. The mean values after 1 s of the applied force can be seen in Figure 13, along with the error bars defined via standard deviations. The value after a 15 ± 1 s time interval was also measured, but this value typically decreased by only 1 Shore D (Sh D) unit.

The same tendency to recover the original parameters is also observable here. Nevertheless, original values were not completely achieved again. The specimens were relatively soft and pliable in some areas right after they were removed from the climate chamber, due to the process of plasticization [46]. Thus, the standard deviation of the measured hardness is significantly higher for those specimens. The standard deviations of the specimens after a few days of relaxation are comparable with the original values measured before the exposure.

Overall, these findings on changes in various mechanical parameters are in a good alignment with outcomes of similar studies [37,49,50]. In this study, it can also be observed that the change in mean values of the yield strength (the maximum stress a material can endure before plastic deformation) and hardness, compared to their original values, behave similarly. The measurement response before the exposure, right after the exposure, and after different relaxation times shows approximately the same relative change as the original value. Thus, these mechanical properties for material degradation evaluation might be interchangeably interpreted, at least in this particular case of unmodified epoxy resin. The mechanical properties and mentioned phenomena are summarized in Table 3.

### 3.2. Degradation Models

The material properties were evaluated and the experimental data were collected and analyzed. Subsequently, the degradation was modeled via different approaches to identify the most suitable one. This degradation model helps us to better understand material behavior under a particular stress or set of stresses and might be further utilized in accelerated-aging experiments in order to obtain an aging model for estimating the remaining useful life in service life evaluations.

The effect of the hygrothermal stress was evaluated via various approaches. These were as follows: (i) physically informed empirical relation, (ii) Response Surface Methodology, and (iii) Bayesian Model Averaging. The same dielectric strength $E_{p}$ testing dataset (presented in Figure 10) was used as the input for all of the aforementioned methods.

#### 3.2.1. Physically Informed Empirical Relation

**Figure 14:**
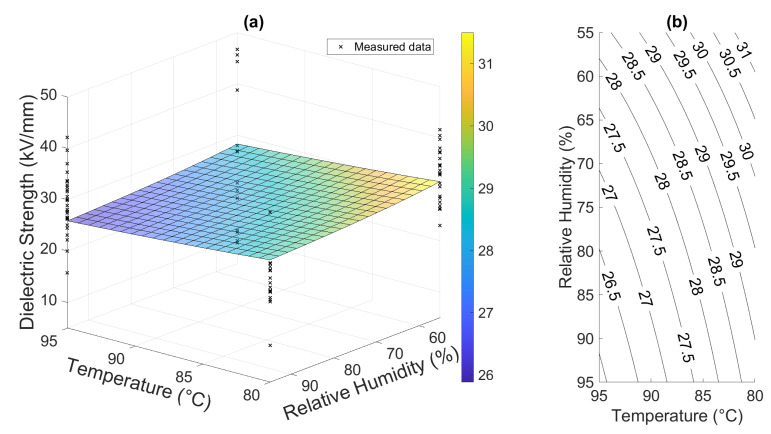
Physically informed empirical model results: (**a**) surface plot of the material response and (**b**) contour plot of the response.

The empirical relation used in the aging model of combined stressing via temperature and relative humidity was employed according to Equation (1) and [21]. Although this relation is used for lifespan evaluations, the formula contains suitable components for the description of degradation. However, the model is rather conservative, showing only slight changes in the prediction of the system’s response to stress in this case. The system response and related contour plot can be seen in Figure 14. The model was built and plotted via the MATLAB R2020a script.

This approach is the simplest one used in this study and does not provide any further information other than surface regression in the experimental space. It can be noted that it is also poorly performing. Therefore, other approaches were utilized.

#### 3.2.2. Response Surface Methodology

**Figure 15:**
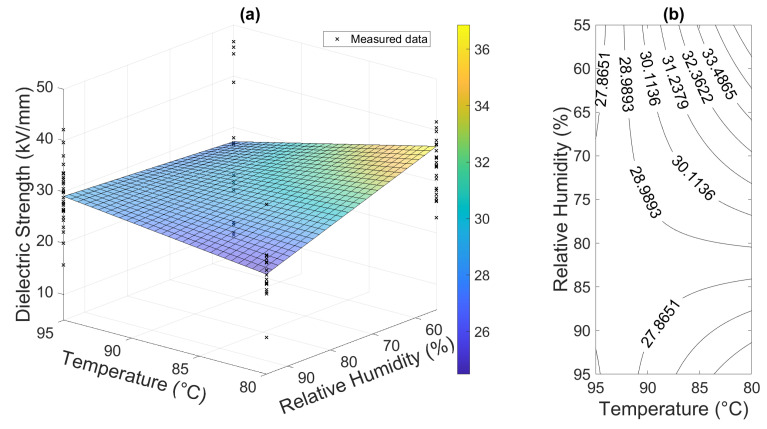
RSM first-order model results: (**a**) response surface of the material and (**b**) contour plot of the response surface.

A well-established engineering procedure, RSM provides a general approach for evaluating the response of a system within any kind of similar factorial experiment [25,26,27]. It is obvious that the curvature of the surface response is more significant compared to the physically informed empirical relation. The surface response is depicted in the form of surface and contour plots in Figure 15. The model was also built and plotted via the MATLAB R2020a script in this case.

**Figure 16:**
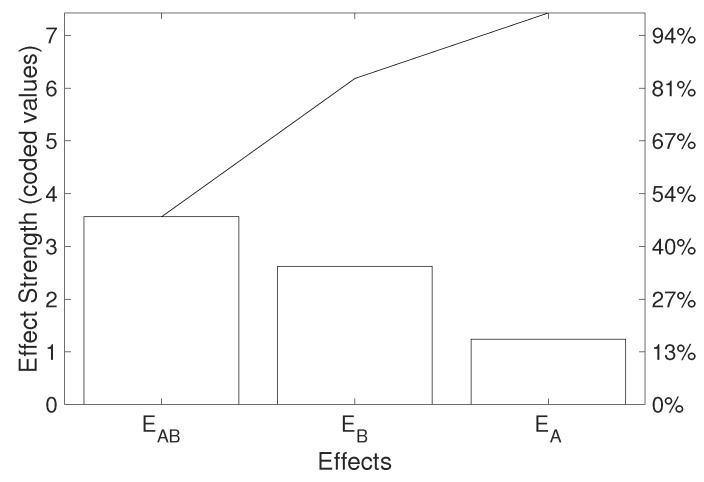
Pareto analysis of the effects obtained via the RSM two-factor first-order model (the black line represents the cumulative percentage).

Moreover, this approach provides information on the strength of different effects according to the RSM. The Pareto analysis shown in Figure 16 was used as a quick tool for such an evaluation. This particular model reported the interaction of relative humidity and temperature as the most significant factor, followed by relative humidity and temperature, respectively. The temperature alone had the least/a minor effect on the degradation according to this particular experiment based on the Pareto principle. There is no need to omit this effect in the model since a two-factor model is simple enough. However, this simple analysis could be useful for the identification of negligible effects in experiments employing a higher number of factors.

#### 3.2.3. Bayesian Experimental Design

**Figure 17:**
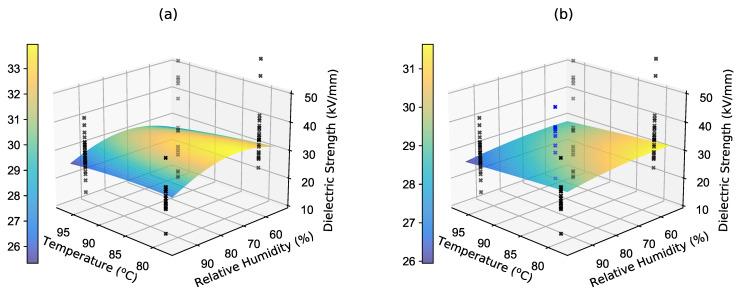
Mean values of the BMA model: (**a**) four points in the experimental space and (**b**) five points in the experimental space (the blue crosses represent the data of the measured fifth point).

**Figure 18:**
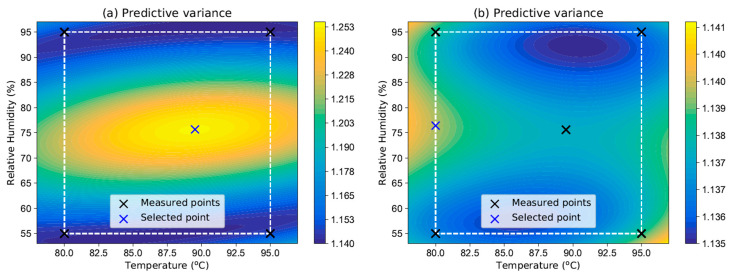
Predictive variance: (**a**) four points in the experimental space and (**b**) five points in the experimental space.

A custom Python implementation of this method was used for the Bayesian model selection. The procedure was carried out in several steps. First, four points selected via a simple first-order DoE procedure associated with the RSM approach were fed into the algorithm, four models (6) were fitted, and their probabilities were calculated. Based on this probability and the dielectric strength predictions for the new points, a BMA predictor (16), as depicted in Figure 17a, was created. The prediction variance (18) shown in Figure 18a was then used as an acquisition function for the new (fifth) measured point. This was located at a temperature of 89.5 °C and at 75.6% relative humidity.

This point was then measured and once again inserted into the algorithm along with the previous four points. The new BMA prediction mean is depicted in Figure 17b and the predictive variance is shown in Figure 18b.

**Figure 19:**
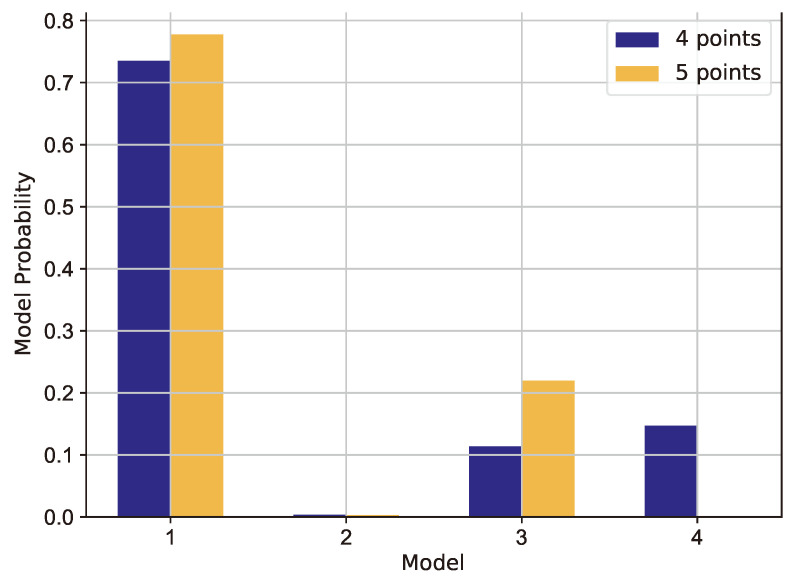
Probabilities of the different Bayesian linear models.

The probability of each model before and after the insertion of the fifth point can be seen in Figure 19 and in Table 4. The first model proved to be a good candidate right from the beginning of the experiment and its probability was further increased by adding a fifth point. The probability of the third model also increased and the fourth model dropped to almost zero. The conclusion could be that the second-order factor terms could be beneficial for increasing the accuracy of the model. The first model might be preferred because of its simplicity.

#### 3.2.4. Model Comparison

The worst prediction was reported using the physically informed empirical relation, usually applied as an aging model for lifespan estimation for similar stress factors. The RSM returned a result that was a bit closer to the one obtained from the validation. The RSM approach was subsequently improved via the implementation of regression enhanced by the Bayesian theorem, where the prior and data coefficients were used for its optimization, which was carried out within the BMA model. This led to a reduction in the curvature of the response surface and the result being even closer to the validation measurement. Regardless, the best prediction of the system response in this study was provided by the BMA model. All respective results and the results of the validation measurement are summarized in Table 5.

## 4. Conclusions

The thermal analyses were performed during the specimen’s manufacturing process and characterization. STA was used in order to investigate the behavior of the tested material in terms of heat flow and mass change in a wide temperature range, which included the operating temperature range and ended with a complete thermal decomposition of the material. An analysis via DSC led us to the determination of a multi-step curing process and an under-cured state under one of the manufacturers’ recommended curing procedures (140 °C for 6 h). However, the under-cured state is usually the delivered condition of epoxy in such an EIS. Therefore, we decided to proceed with these specimens. The main goal here was to evaluate this specimen’s manufacturing procedure, explore parameters, and determine the limit where thermal degradation is not present in this particular material.

A hygrothermal degradation experiment on an impregnating mono-component epoxy resin was carried out. The effect of this degradation process was observed via dielectric strength testing. This effect was also statistically evaluated and confirmed along with similarly oriented studies. The degradation effect was also confirmed via mechanical measurements of tensile force and Shore D hardness, carried out after relaxation for various time intervals. The original state of the specimens was not achieved even after 4 days within the laboratory’s ambient conditions. The degradation effect was also reported using a vector bridge and BDS measurements. Both of the aforementioned methods proved the significant effect of hygrothermal degradation as the permittivity and dissipation factors changed greatly. However, longer hygrothermal exposures and the effect of the samples drying should be investigated in future work as these were not incorporated into this study. The moisture uptake might be measured by means of gravimetric techniques, as suggested in [46,47], in order to perform better-controlled hygrothermal degradation experiments.

The results of the dielectric strength measurement were subsequently used in order to obtain the degradation models. Several degradation evaluation approaches were introduced and carried out. The Bayesian Model Averaging (BMA) model was identified as superior, as its prediction was the closest one to the validation measurement. The physically informed empirical model performed the worst, with an error of almost 17%, while the BMA reported only about 2.5% of difference compared to the validation measurement in this study. Moreover, the BMA approach proposes two significant benefits: (i) the retrieval of the suitable model in as few steps as possible, and (ii) the suggestion of the next point in the experimental space based on the predictive variance. This approach suggests a promising method for planning degradation experiments and evaluating them.

The possibility of interchanging the yield strength (also considered within [1]) and the hardness as a system response to degradation is proposed for materials similar to an unmodified mono-component epoxy resin, since these parameters changed proportionally in the same manner. Thus, it might be of interest to investigate the degradation effect via one of these responses. The degradation of various mechanical parameters is also reported within the outcomes reported in [37,49,50]. Even though the dielectric strength is recommended in [1], it is not advised to strictly focus on this parameter based on the data acquired in this study, as it typically has a high variance and less significant difference in its mean values across the respective groups of specimens. The dissipation factor or relative permittivity change could also be a suitable response to degradation, as indicated in [37] or [51].

## Figures and Tables

**Table 1:** The experimental plan: settings of the degradation factors.

Setting No.	Temperature (°C)	Relative Humidity (%)
#1	95 (high)	95 (high)
#2	95 (high)	55 (low)
#3	80 (low)	95 (high)
#4	80 (low)	55 (low)

**Table 2:** Dielectric properties of the tested specimen before and after hygrothermal stressing, measured via a vector bridge.

	Rel. Permittivity	Dissipation Factor	Capacity
	$\epsilon_{r}$ **(-)**	tanδ **(-)**	$C_{x}$ **(pF)**
Before exposure	2.882	0.00367	38.170
After exposure	4.225	0.04800	55.800

**Table 3:** Mechanical properties of hydrothermally stressed samples: before exposition, right after exposition, and after relaxation, including differences in the properties compared to the original values before the stress.

	Yield Strength	Difference	Hardness Sh D	Difference
	$\sigma_{Y}$ **(MPa)**	$\Delta \sigma_{Y}$ **(%)**	$S_{D}$ **(-)**	$\Delta S_{D}$ **(%)**
Before	56.19	0.00	80.8	0.00
Right after	37.17	33.84	71.1	12.00
1-day relaxation	39.86	29.07	73.1	9.53
4-days relaxation	48.96	12.87	77.0	4.70

**Table 4:** Model probabilities before and after the addition of the fifth point.

Model	4 Points	5 Points
$M_{1}$	0.74	0.78
$M_{2}$	$10^{-3}$	$10^{-3}$
$M_{3}$	0.11	0.22
$M_{4}$	0.15	$10^{-7}$

**Table 5:** Comparison of different degradation models and their validation measurement results.

Model	Validation Estimation $E_{p}$ (kV/mm)	Validation Result $E_{p}$ (kV/mm)
Physically informed empirical	27.67	33.31
First-order RSM	29.05	
Bayesian first-order RSM	30.52	
BMA (multiple candidates)	32.52	

## Data Availability

The original contributions presented in the study are included in the article, further inquiries can be directed to the corresponding author.

## Abbreviations

The following abbreviations are used in this manuscript:

EIS	electrical insulation system
CMD	Condition Monitoring and Diagnostics
CbM	Condition-based Maintenance
PdM	Predictive Maintenance
DoE	design of experiments
RSM	Response Surface Methodology
ANN	Artificial Neural Network
SVM	Support Vector Machine
ANOVA	Analysis of Variance
STA	Simultaneous Thermal Analysis
DSC	Differential Scanning Calorimetry
TGA	Thermogravimetric Analysis
DMA	Dynamic Mechanical Analysis
BDS	Broadband Dielectric Spectroscopy
BMA	Bayesian Model Averaging
RH	relative humidity
